# Supplementary material for: Anomalous isotope effect on mechanical properties of single atomic layer Boron Nitride
Source: Nat Commun. 2023 Sep 1;14:5331. doi: 10.1038/s41467-023-41148-2 (PMC10474280; doi:10.1038/s41467-023-41148-2)
Supplement: Supplementary file 1 — Supplementary Information [file 41467_2023_41148_MOESM1_ESM.pdf]

Supplementary Information

Anomalous Isotope Effect on Mechanical Properties of Single Atomic Layer Boron Nitride

Alexey Falin,[1] Haifeng Lv,[2] Eli Janzen,[3] James H. Edgar,[3] Rui Zhang,[4] Dong Qian,[4] Hwo-Shuenn Sheu,[5] Qiran Cai,[1] Wei Gan,[1] Xiaojun Wu,[2] Elton J. G. Santos,[6,7] Lu Hua Li[1]*

1. Institute for Frontier Materials, Deakin University, Waurn Ponds Campus, Waurn Ponds, VIC 3216, Australia
2. Hefei National Laboratory for Physical Sciences at the Microscale, School of Chemistry and Material Sciences, CAS Key Laboratory of Materials for Energy Conversion, and CAS Center for Excellence in Nanoscience, University of Science and Technology of China, Hefei, Anhui 230026, China
3. Tim Taylor Department of Chemical Engineering, Kansas State University, Manhattan, Kansas 66506, USA
4. Department of Mechanical Engineering, The University of Texas at Dallas, Richardson, Texas 75080, USA
5. National Synchrotron Radiation Research Center, Hsinchu 300, Taiwan
6. Institute for Condensed Matter Physics and Complex Systems, School of Physics and Astronomy, The University of Edinburgh, EH9 3FD, United Kingdom.
7. Higgs Centre for Theoretical Physics, The University of Edinburgh, EH9 3FD, United Kingdom.

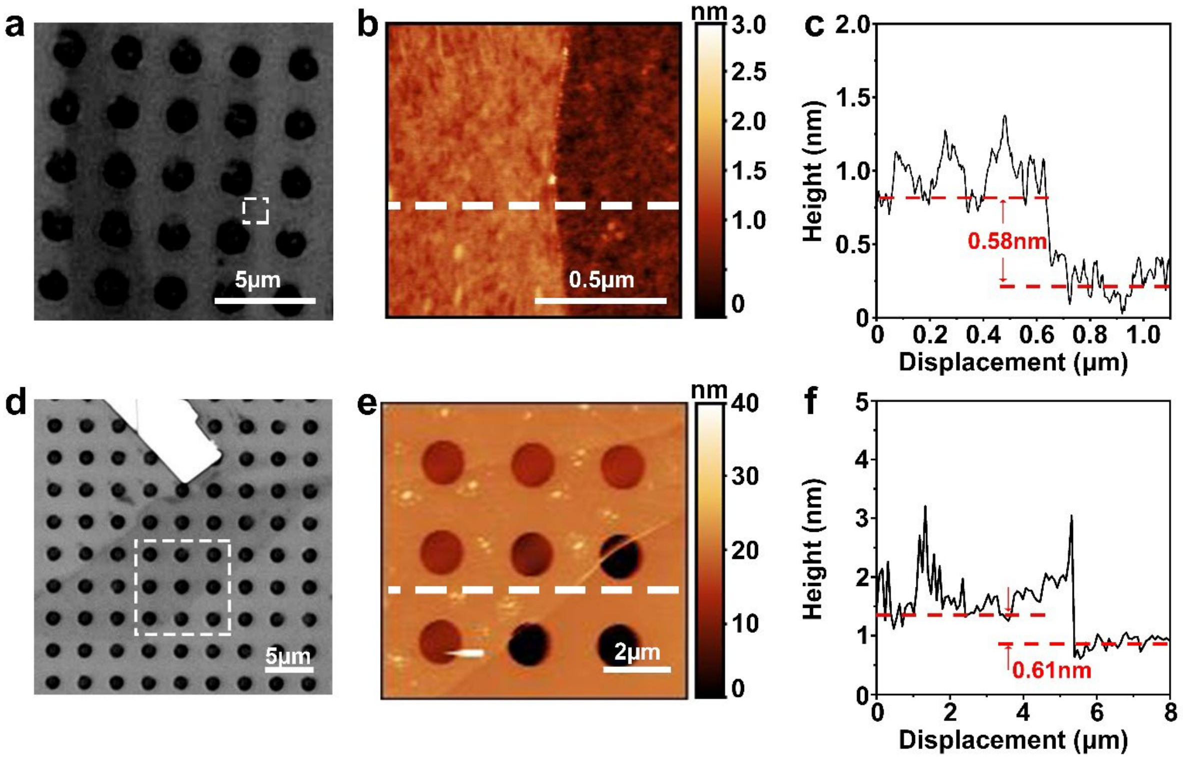

Supplementary Fig. 1 | Characterization of BN nanosheets. Optical microscopy images of **a,** a 1L ^{Nat}BN and **d,** a 1L ^{10}BN on a SiO_2/Si substrate with micro-wells of 1.6 μm in diameter. Repulsive tapping mode AFM images of **b,** the ^{Nat}BN nanosheets and **e,** the ^{10}BN nanosheets marked in the square of (**a**) and (**d**), respectively. **c,** and **f,** the corresponding height traces of the dashed lines for ^{Nat}BN and ^{10}BN samples, respectively, confirming the monolayer thickness.

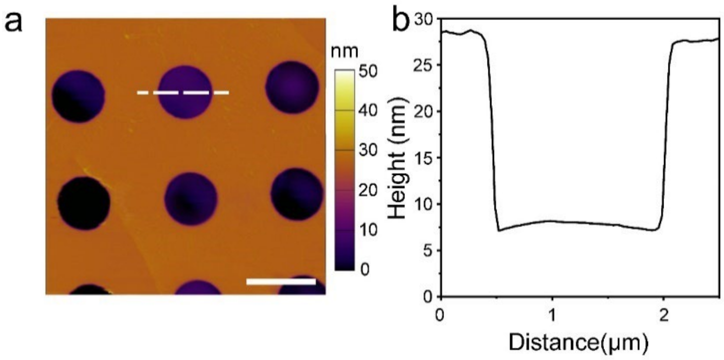

Supplementary Fig. 2 | Characterization of a suspended region of a monolayer BN. a, Repulsive tapping mode AFM image of the ^{11}BN nanosheet. **b,** the corresponding height trace of the dashed line in (**a**) representing the suspended part of the monolayer BN.

Supplementary Note 1: ***Measurements of mechanical properties of 2D nanomaterials using AFM***

Atomic force microscope (AFM) has been the mostly used method to measure the mechanical properties of 2D materials[1,4-6] due to the ease of accessibility as well as high reliability, though other methods such as in-plane stretching[7,8] and bulge method[9,10] have also been demonstrated. The AFM method was first used on graphene by C. Lee et al. (2008)[1]. In the method, the 2D materials are suspended over microwells or trenches pre-fabricated on substrates. Importantly, the 2D sheets must be securely attached to the edge of the microwell or trench to prevent sliding during indentation. The AFM cantilever with a sharp tip at its end applies loads at the centre of suspended regions of the 2D materials. The material of the indenter or AFM tip is ideally stiffer than the 2D material (e.g. diamond tip for graphene and BN measurement). Also, the tip radius should be much smaller than the radius of the suspended 2D materials. The load-displacement relations provide information on the elastic properties and fracture force of the 2D materials.

To determine the Young's modulus, Eq. (1) is used, which is based on a combination of the solutions from 1) the linear behavior of 2D materials under small stress or indentation depths derived by Wan et al.[11], and 2) for large stresses, where load varies as the cube of displacement derived by Komaragiri et al.[12]:

$$F(\delta) = \pi {\sigma_0}^{2D} \delta + \left(\frac{q^3 E^{2D}}{a^2} \right) * \delta^3, \qquad (1)$$

This equation is applied to fit the load-displacement $F(\delta)$ curves from AFM indentation. The first linear term of the load-displacement curve depends only on 2D pre-tension (${\sigma_0}^{2D}$) due to axial tensions in the 2D materials, given fixed radii of the tip and the suspended 2D materials (a). In this linear region, the initial load and small stretch flatten the suspended 2D materials by removing wrinkles and unevenness. The pre-tension value mostly depends on the sample itself and its preparation process. This term could also include bending modulus, but this factor is negligible at the atomic thickness. With further increase in the load, the second term of Eq. (1) starts to play an important role. The load–displacement relation becomes cubic dependent and reflects the stiffness of the suspended 2D materials so that the 2D Young's modulus (E^{2D})

value can be obtained. This term also depends on Poisson's ratio (υ) of the 2D material which is included in Eq. (1) as a dimensionless constant $q = 1/(1.05 - 0.15\upsilon - 0.16\upsilon^2)$. In this analysis, only pre-tension and Young's modulus are variables in fitting the load-displacement curves. Later, this equation was further developed by Lin et al.(2013)[13]. The updated relation considers starting (zero) coordinates (f_0 and δ_0) as additional variables that could improve the accuracy of the calculated Young's modulus by removing the manual determination of the zero point, i.e. Eq. (1) in main text. The accuracy of the elastic properties from the load-displacement data fitting relies on whether the curve has reached the transformed Schwerin (cubic) term[14] in Eq. (1) (main text)[15]. High-quality graphene and 2D BN can typically withstand significant levels of strain to reach this region without failure.

To obtain the fracture strength of 2D materials, the measured elastic modulus, fracture load, and load-displacement curves are normally analysed by finite element method (FEM), as the strength value calculated analytically is overestimated due to the lack of nonlinear component, which is especially critical under high loads prior fracture.

The above AFM indentation method and analysis of the load-displacement curves have been successfully applied for graphene and other 2D materials, such as BN and transition metal dichalcogenides (TMDCs)[1,6]."

Supplementary Note 2: ***Uncertainty Analysis and t-Test statistics.***

The displacement accuracy of the Cypher AFM was estimated by calibrating the z-displacement of the piezoelectric stage using standard samples of known height, i.e. a CalibratAR grating. This standard sample had rectangular SiO_2 steps on a Si wafer with the certified step height of 200±4 nm. The chosen grating height was similar to the characteristic indentation displacement. The standard sample was scanned in several locations, using contact mode. The obtained step height was 201.4±0.1 nm in average with a relative error of ~0.05%. The expanded uncertainty of the difference between the measured results and certified height value is higher than the absolute difference between the mean measured value and certified height value, indicating that there was no significant difference between the measured mean and certified height value. Therefore, the systematic error of displacement measurements was considered negligible compare to random errors of the measured displacement. The

measurement accuracy of displacement is 0.7%. To estimate the uncertainty of the force measurements $F = k{\cdot}\delta$, the accuracy of the cantilever spring constant was needed. The spring constants were estimated by a combination of two methods: Sader and the thermal noise methods. All cantilevers used in the experiment had a high stiffness k of >40 N m^{-1}. The estimated uncertainty of the spring constant measurement was ~2.6%. This gave rise to the force measurement uncertainty of 2.7%. Although photodiode sensitivity also contributes to the uncertainty of the Young's modulus estimation, in our case the photodiode sensitivity value was small ~1 and considered not contributing to the systematic error. The uncertainty of the Young's modulus depended on applied load, indentation displacement, and the radius of the suspended atomically thin BN. The uncertainty in the measurements of the membrane radius was negligible as suspended regions are quite large compare to the error of lateral AFM positioning. So the uncertainty of Young's modulus is:[1]

$$\frac{\Delta E}{E} = \sqrt{\left(\frac{\Delta F}{F}\right)^2 + \left(3\frac{\Delta \delta}{\delta}\right)^2} \qquad (2)$$

This gave the Young's modulus uncertainty of 3.4%, *i.e.* systematic error values ±10.2 Nm^{-1} and ±9.6 Nm^{-1} for the 1L ^{10}BN and ^{11}BN, respectively. The uncertainty of the fracture strength measurements can be estimated:

$$\frac{\Delta \sigma}{\sigma} = \sqrt{\left(\frac{\Delta F}{2F}\right)^2 + \left(\frac{\Delta E}{2E}\right)^2} \qquad (3)$$

where the uncertainty of the tip radius was assumed to be small as measured by TEM, and non-linear contribution to the force has a scale factor of ~0.1, which has a negligible influence on strength accuracy value. Eq. (4) yielded the uncertainty of fracture strength to be ~3.2%. Therefore, the systematic error values were ±0.81 Nm^{-1} and ±0.78 Nm^{-1} for the 1L ^{10}BN and ^{11}BN, respectively.

In the experiment, we measured the mechanical properties of 24 samples of 1L ^{10}BN and 23 samples of ^{11}BN. The statistics on the experimental results is in the Supplementary Table 1 below:

Supplementary Table 1. Means with standard deviations and standard errors of the Young's modulus and fracture strength of ^{Nat}BN, ^{10}BN and ^{11}BN monolayers.

Sample	x	Parameter	N	Mean [N m^{-1}]	s_x [N m^{-1}]	Variance [N^2 m^{-2}]	SE [N m^{-1}]
1L ^{10}BN	1	Young's modulus	24	298.66	22.20	493.06	4.53
		Fracture strength	24	25.30	2.45	6.00	0.50
1L ^{Nat}BN	2	Young's modulus	45	284.25	20.83	433.92	3.11
		Fracture strength	45	24.60	2.47	6.09	0.37
1L ^{11}BN	3	Young's modulus	23	281.23	10.04	100.75	2.09
		Fracture strength	23	24.41	1.74	3.04	0.36

where, s_x – standard deviation, SE –standard error.

To obtain the full measurement error, the random and systematic errors were combined. The Young's modulus full standard deviation and estimated mean values were $\bar{\mu}_1$ =298.66±24.42 N m^{-1} and $\bar{\mu}_3$ =281.23±13.86 N m^{-1} for 1L ^{10}BN and 1L ^{11}BN, respectively. The fracture strength full standard deviation and estimated mean values were $\bar{\sigma}_1$ =25.3±2.58 N m^{-1} and $\bar{\sigma}_3$ =24.4±1.91 N m^{-1} for 1L ^{10}BN and 1L ^{11}BN, respectively. To determine the significance of the difference in the mechanical values of ^{10}BN and ^{11}BN monolayer samples, the two-sample t-Test with unequal variances was used. For Young's modulus values of 1L ^{10}BN and 1L ^{11}BN, we tested the hypothesis that two samples have equal means. The mean values $\bar{\mu}_1$ =298.66 N m^{-1} and $\bar{\mu}_3$ =281.23 N m^{-1}, for 1L ^{10}BN and 1L ^{11}BN, respectively. The standard deviation s_x was determined by:

$$s_x = \sqrt{\frac{\sum_{i=1}^{N}(X_i - \bar{\mu}_x)^2}{N-1}} \qquad (4)$$

where, x is the sample number (x=1 is 1L ^{10}BN and x=3 is 1L ^{11}BN), X_i and $\bar{\mu}_x$ – Young's modulus values of individual measurements (*i*) and mean value of sample x, N-number of measurements. From this, the statistic T is:

$$T = \frac{\bar{\mu}_1 - \bar{\mu}_3}{\sqrt{\left(\frac{s_1^2}{N_1} + \frac{s_3^2}{N_3}\right)}} \qquad (5)$$

The degrees of freedom (*df*) for t-distribution was found using the Welch–Satterthwaite equation:

$$df = \frac{\left(\frac{s_1^2}{N_1} + \frac{s_3^2}{N_3}\right)^2}{\frac{s_1^4}{N_1{}^2(N_1 - 1)} + \frac{s_3^4}{N_3{}^2(N_3 - 1)}} \qquad (6)$$

The two-tailed T-test statistics was applied, as the mean Young's modulus of the 1L ^{10}BN was greater than that of the 1L ^{11}BN, i.e. $\bar{\mu}_1 > \bar{\mu}_3$ to test the null hypothesis that the mean values of the Young's modulus of 1L ^{10}BN and 1L ^{11}BN were equal. The same operation was performed with fracture strength values.

Supplementary Table 2. The T-test values and results.

Sample	x	Parameter	N	Mean [N m^{-1}]	FE [N m^{-1}]	Variance [N^2 m^{-2}]	SE [N m^{-1}]
1L ^{10}BN	1	Young's modulus	24	298.66	24.42	596.16	4.98
		Fracture strength	24	25.30	2.58	6.65	0.53
1L ^{11}BN	3	Young's modulus	23	281.23	13.86	192.18	2.89
		Fracture strength	23	24.41	1.91	3.65	0.40

Parameter	T value	df	Tcritical	p-value
Young's modulus	3.024	36	2.028	0.005
Fracture strength	1.338	42	2.018	0.188

The T statistics value was compared to the T-Critical value of the two-tail statistic. Since the p-value was smaller than the significance level (α) of 0.05, the null hypothesis was rejected for the Young's modulus data, as there was a significant difference in Young's modulus means between tested samples. In the case of fracture strength values, the null hypothesis could not be rejected.

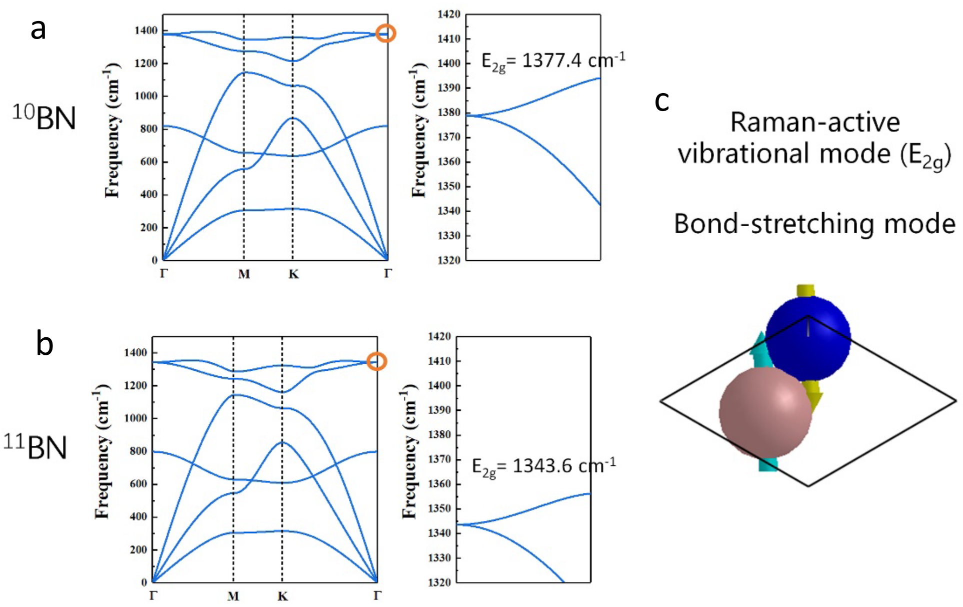

Supplementary Fig. 3 | Phonon Spectra-BN monolayer. Phonon spectra of **a,** ^{10}BN **b,** ^{11}BN, and **c,** diagram showing the E_{2g} mode in hBN.

Supplementary Table 3. Lattice and elastic constants and Young's moduli for 2×2 supercell of BN monolayer (Figure 3a) biaxial stretching from DFT at 0 K. For hexagonal BN monolayer, C11 = C22, $E = \frac{Y^{2D}}{0.334}$, $Y^{2D} = \frac{C11*C22-C12^2}{C11}$.

Monolayer	a (Å)	C11 (N m^{-1})	C12 (N m^{-1})	C66 (N m^{-1})	Y_{2D} (N m^{-1})	E (GPa)
^{10}BN	2.51111	294.964	66.198	114.383	280.1	838.6
^{11}BN	2.51278	293.705	65.625	114.040	279.0	835.3

Supplementary Table 4. Total (Etot) [eV] and bond dissociation (Eb) energies [eV per unit cell] of interatomic interaction from for 2×2 supercell of BN monolayer (Figure 3a) at 0 K.

Monolayer	a (Å)	Area (Å^2)	Etot	E_Boron	E_Nitrogen	Eb	Eb/A
^{10}BN	2.51111	5.46095	-17.71984	-3.21811	-2.80372	-11.69801	-2.14212
^{11}BN	2.51278	5.46815	-17.71980	-3.21274	-2.80372	-11.70334	-2.14027
Δ (^{11}B-^{10}B)	0.00167	0.0072	0.00004	0.00537	0	-0.00533	0.00185

Supplementary Table 5. Lattice and Young's moduli for 2x2 supercell of BN monolayer (Figure 3a) biaxial stretching from DFT at room temperature.

Monolayer	a (Å)	Y_{2D} (N m^{-1})	E (GPa)
^{10}BN	2.51864	279.1	835.6
^{11}BN	2.51906	276.7	828.4

Total (Etot) [eV] and bond dissociation (Eb) energies [eV per unit cell] of interatomic interaction from for 2x2 supercell of BN monolayer (Figure 3a) at 300 K.

Supplementary Table 6

Monolayer	a (Å)	Area (Å^2)	Etot	E_Boron	E_Nitrogen	Eb	Eb/A
^{10}BN	2.51864	5.49369	-17.42186	-3.21811	-2.80372	-11.40003	-2.07512

^{11}BN	2.51906	5.49551	-17.41891	-3.21274	-2.80372	-11.40246	-2.07487
Δ (^{11}B-^{10}B)	0.00042	0.00182	0.00295	0.00537	0	-0.00243	0.00025

Supplementary Table 7. Lattice constants of isotopic BN powder samples were measured by the synchrotron XRD technique.

Name	Sample	Unit cell constants (BN)
^{11}BN powder	^{11}BN Powder	a=2.5051(6)<br>c=6.6651(13)
^{10}BN powder	^{10}BN Powder	a=2.5038(2)<br>c=6.6552(3)

Supplementary Note 3: ***Interatomic potential***

In the simple diatom case of the potential energy of B−N bond, the zero-point vibrational state can be defined as the lowest energy state of a quantum harmonic oscillator (QHO) system. This implies that atoms in the system are not at rest at 0 K and it is related to the Heisenberg uncertainty principle.[2] The zero-point vibrational state can be found as:

$$V_0 = U_0 + \frac{1}{2}\hbar w_0 \qquad (7)$$

where, U_0 is the minimum energy of the interatomic classical potential, $\hbar$ - reduced Planck's constant, w_0 - the angular oscillation frequency of QHO. In the case of the interatomic bond, two atoms oscillate relative to each other and their oscillation frequency depending on their mass and the nature of the bond:

$$w_0 = \sqrt{\frac{S_0}{\mu}} = \sqrt{\frac{S_0(m_1 + m_2)}{m_1 m_2}} \qquad (8)$$

where S_0 is a stiffness constant of the interatomic bond, μ – reduced mass, and actual m_1 and m_2 masses of the interacting atoms. By combining Eq. (7) and Eq. (8) it can be seen how masses of the interacting atoms affect the zero-point vibrational energy level:

$$V_0 = U_0 + \frac{1}{2}\hbar\sqrt{\frac{S_0(m_1 + m_2)}{m_1 m_2}} \qquad (9)$$

By substituting only one kind of atom in a compound on its isotope, it can be seen that the zero vibrational levels will be lower for the heavier isotope. The stiffness of the bond at 0 K can be derived from the potential energy of the bond:

$$E_0 = \frac{S_0}{r_0} = \frac{1}{r_0}\frac{d^2V(r)}{d{r_0}^2} \qquad (10)$$

where E_0 is Young's modulus of the bond; r_0 is an equilibrium interatomic distance at 0 K; $V(r)$ is potential energy between the interacting atoms at the distance r.

With increased temperature, the change in the energy can be estimated as:[3]

$$V(r) = V_0 + 3\kappa N_A T \cdot D(\frac{T_\theta}{T}) \qquad (11)$$

where N_A is the Avogadro constant κ is the Boltzmann constant, T is temperature, T_θ is the Debye temperature, and $D(x)$ is the Debye function:

$$D(x) = \frac{3}{x^3}\int_0^x \frac{z^3 dz}{e^z - 1} \qquad (12)$$

where $z = \frac{T_\theta}{T}$. The relation between Debay temperatures of different isotope samples in the approximation of small and harmonic atoms' oscillations:

$$T_\theta^{11B} = T_\theta^{10B}\sqrt{\frac{m_{10B}}{m_{11B}}} \qquad (13)$$

where m_{11B} and m_{10B} are masses of boron atoms in ^{11}BN and ^{10}BN samples. The difference between energies of vibrational levels for ^{11}BN and ^{10}BN samples:

$$\Delta V_{11-10B} = \Delta V_0 + \Delta V_T \qquad (14)$$

where ΔV_0 is the change in the zero vibrational energy between ^{11}BN and ^{10}BN samples. ΔV_T is a difference in thermal energy contribution:

$$\Delta V_T = 3\kappa \mathrm{N_A} T \cdot \left[D\left(\frac{T_\theta^{10B}}{T} \sqrt{\frac{m_{10B}}{m_{11B}}} \right) - D\left(\frac{T_\theta^{10B}}{T} \right) \right] \qquad (15)$$

As $m_{11B} > m_{10B}$ and Debye function monotonically decrease with increasing x, $\Delta V_T > 0$. The thermal energy contribution to the vibrational states of ^{11}BN and ^{10}BN samples will increase with increased temperature, leading to changes in the isotope effect at room temperature compared to 0 K.

Supplementary References:

1 Lee, C., Wei, X., Kysar, J. W. & Hone, J. Measurement of the Elastic Properties and Intrinsic Strength of Monolayer Graphene. *Science* **321**, 385-388 (2008).

2 Heisenberg, W. Über den anschaulichen Inhalt der quantentheoretischen Kinematik und Mechanik. *Zeitschrift für Physik* **43**, 172-198 (1927).

3 Kogan, V. Isotope effects in the structural properties of solids. *Soviet Physics Uspekhi* **5**, 951 (1963).

4 Bertolazzi, S., Brivio, J. & Kis, A. Stretching and breaking of ultrathin MoS_2. *ACS Nano* **5**, 9703-9709 (2011).

5 Falin, A. *et al.* Mechanical Properties of Atomically Thin Tungsten Dichalcogenides: WS_2, WSe_2, and WTe_2. *ACS Nano* **15**, 2600-2610 (2021).

6 Falin, A. *et al.* Mechanical properties of atomically thin boron nitride and the role of interlayer interactions. *Nature Communications* **8**, 15815 (2017).

7 Zhang, P. *et al.* Fracture toughness of graphene. *Nature communications* **5**, 1-7 (2014).

8 Cao, K. *et al.* Elastic straining of free-standing monolayer graphene. **11**, 284 (2020).

9 Nicholl, R. J. T., Lavrik, N. V., Vlassiouk, I., Srijanto, B. R. & Bolotin, K. I. Hidden area and mechanical nonlinearities in freestanding graphene. *Phys Rev Lett* **118**, 266101 (2017).

10 Koenig, S. P., Boddeti, N. G., Dunn, M. L. & Bunch, J. S. Ultrastrong adhesion of graphene membranes. *Nat Nanotechnol* **6**, 543-546 (2011).

11 Wan, K.-T., Guo, S. & Dillard, D. A. J. T. S. F. A theoretical and numerical study of a thin clamped circular film under an external load in the presence of a tensile residual stress. *Thin Solid Films* **425**, 150-162 (2003).

12 Komaragiri, U., Begley, M. & Simmonds, J. J. J. A. M. The mechanical response of freestanding circular elastic films under point and pressure loads. Journal of Applied Mechanics **72**, 203-212 (2005).

13 Lin, Q.-Y. *et al.* Stretch-Induced Stiffness Enhancement of Graphene Grown by Chemical Vapor Deposition. *ACS Nano* **7**, 1171-1177 (2013).

14 Schwerin, E. Über Spannungen und Formänderungen kreisringförmiger Membranen. *ZAMM - Journal of Applied Mathematics and Mechanics / Zeitschrift für Angewandte Mathematik und Mechanik* **9**, 482-483 (1929).

15 Vella, D. & Davidovitch, B. Indentation metrology of clamped, ultra-thin elastic sheets. *Soft Matter* **13**, 2264-2278 (2017).
